# Delayed emergence with remimazolam after laparoscopic subsegmental hepatectomy including the intermittent pringle maneuver in a patient with preserved liver function

**DOI:** 10.1186/s40981-026-00847-7

**Published:** 2026-02-05

**Authors:** Reina Miyazaki, Satoru Sekiya, Hiromu Okano, Shunsaku Goto, Hiroshi Okamoto

**Affiliations:** 1 Department of Critical Care Medicine, St. Luke’s International Hospital, 9-1 Akashi-cho, Chuo-ku, Tokyo 104-8560 Japan; 2 Department of Social Medical Sciences, Graduate School of Medicine, International University of Health and Welfare, 4-1-26 Akasaka, Minato City, Tokyo 107-8402 Japan

**Keywords:** Remimazolam, Delayed emergence, Pringle z, Subsegmental hepatectomy

**To the Editor**

Remimazolam is a novel, short-acting benzodiazepine first introduced for general anesthesia since 2020 and adopted for procedural sedation in many countries [1]. This drug is primarily and rapidly hydrolyzed by hepatic carboxylesterase 1 into an inactive carboxylic acid metabolite. Although remimazolam generally exhibits favorable pharmacokinetics due to its rapid metabolism, pharmacokinetic modeling has demonstrated that its context-sensitive half-time increases with prolonged infusion, indicating that some accumulation does occur over time [2]. Accordingly, delayed emergence from remimazolam anesthesia has been reported [3]. We report a case of a patient with normal preoperative liver function who underwent laparoscopic subsegmental hepatectomy using the intermittent Pringle maneuver. Altered consciousness, attributed to remimazolam, necessitated postoperative intensive care unit (ICU) admission under mechanical ventilation.

An 85-year-old man (height, 169.0 cm; weight, 75.0 kg) with a history of cholecystectomy was scheduled for laparoscopic extended subsegmental hepatectomy for hepatocellular carcinoma. The preoperative examination revealed normal liver and renal function.

General anesthesia was administered using remimazolam and remifentanil with epidural anesthesia. General anesthesia was maintained with 0.7 mg/kg/h of remimazolam and 0.05 µg/kg/min of remifentanil, and the patient state index remained approximately 20–25. During the hepatectomy, the intermittent Pringle maneuver was performed seven times for a total duration of 105 min, involving resection of 7.2% of the liver. The surgery time was 6 h and 44 min. Remimazolam was reduced to 0.3 mg/kg/h 15 min before the procedure and discontinued at completion of the procedure (total dose: 382 mg).

Two hours after discontinuation of remimazolam, the patient had not regained consciousness despite intravenous administration of *flumazenil 1.0 mg* and was admitted to the ICU under mechanical ventilation. Naloxone 0.2 mg was administered for suspected opioid intoxication, but it produced no significant improvement in consciousness. Six hours after discontinuation of remimazolam, infiltration was discovered in the intravenous line used for flumazenil administration, suggesting that the initial postoperative administration may have been ineffective. After re-administration of *flumazenil 0.5 mg*, his consciousness improved dramatically within one minute, from Glasgow Coma Scale E1VTM1 to E4VTM6. His impaired consciousness was thus attributed to remimazolam. After initial improvement, his level of consciousness declined again within one hour. Fifteen hours after discontinuation of remimazolam, his consciousness improved without additional flumazenil, and he was successfully extubated. Twenty-two hours after discontinuation of remimazolam, the patient exhibited somnolence, and *flumazenil 0.5 mg* was readministered for diagnostic purposes, resulting in an improvement in his level of consciousness (Fig. 1). The patient was discharged from the ICU on the following day with an otherwise uneventful postoperative course and preserved renal function, although postoperative day 1 laboratory tests showed elevated liver enzymes with Aspartate Aminotransferase 286 U/L, Alanine Aminotransferase 165 U/L.

**Fig. 1:**
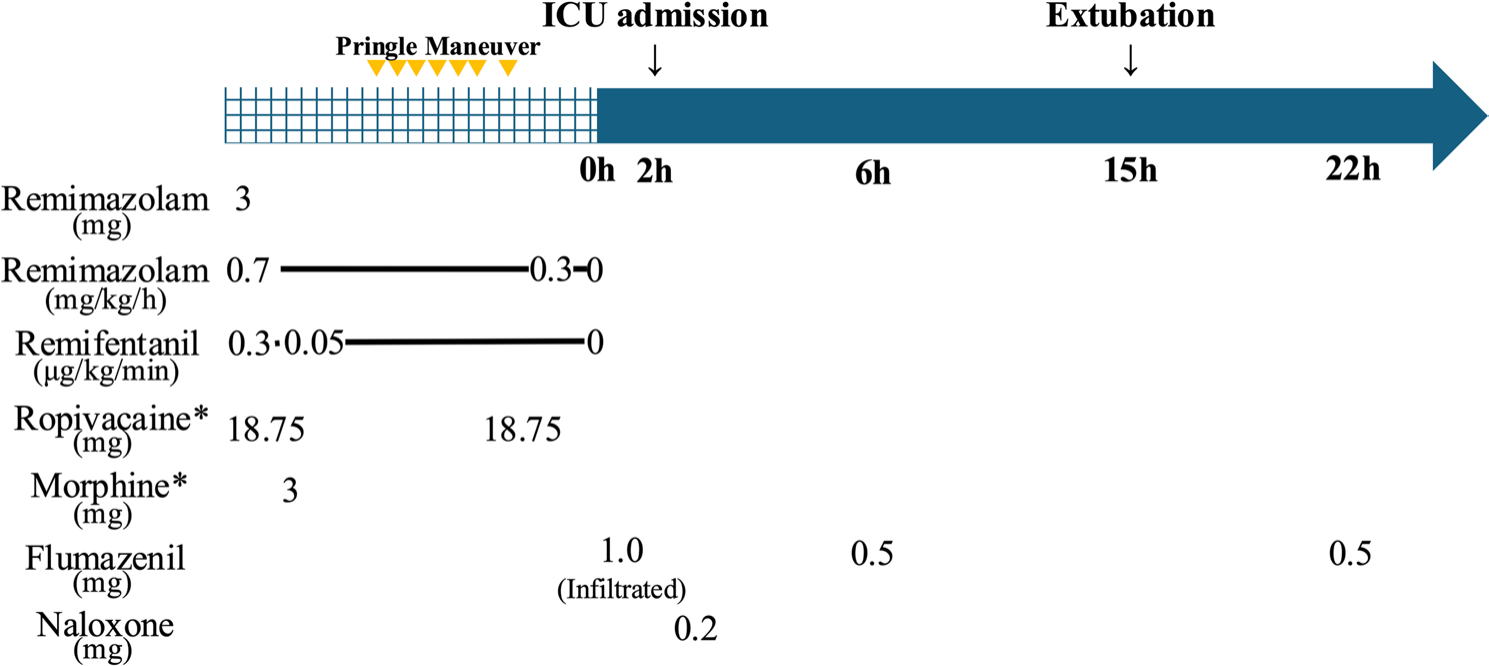
Time course of the perioperative period. The gridded region represents the intraoperative period under general anesthesia. The yellow triangles indicate the timings of the Pringle maneuver. The blue line represents the postoperative course. Asterisk* means epidural infusion

This patient appeared to exhibit delayed emergence attributed to remimazolam. Simulations of effect-site concentrations were performed based on the three-compartment pharmacokinetic model (Fig. 2) [2]. The simulations did not indicate effect-site concentrations sufficient to explain the delayed awakening. However, postoperative liver dysfunction was observed, suggesting that reduced hepatic blood flow during the Pringle maneuver—an effect not reflected in the model—may have impaired remimazolam metabolism. The Pringle maneuver has been associated with ischemia–reperfusion injury accompanied by inflammatory cytokines and oxidative stress [4]. Experimental studies have shown that inflammatory liver injury can suppress hepatic carboxylesterase 1 activity, which is responsible for remimazolam metabolism [5]. Therefore, even in patients with normal preoperative liver function, potential prolongation of remimazolam effects should be considered when surgery involves the Pringle maneuver.

**Fig. 2:**
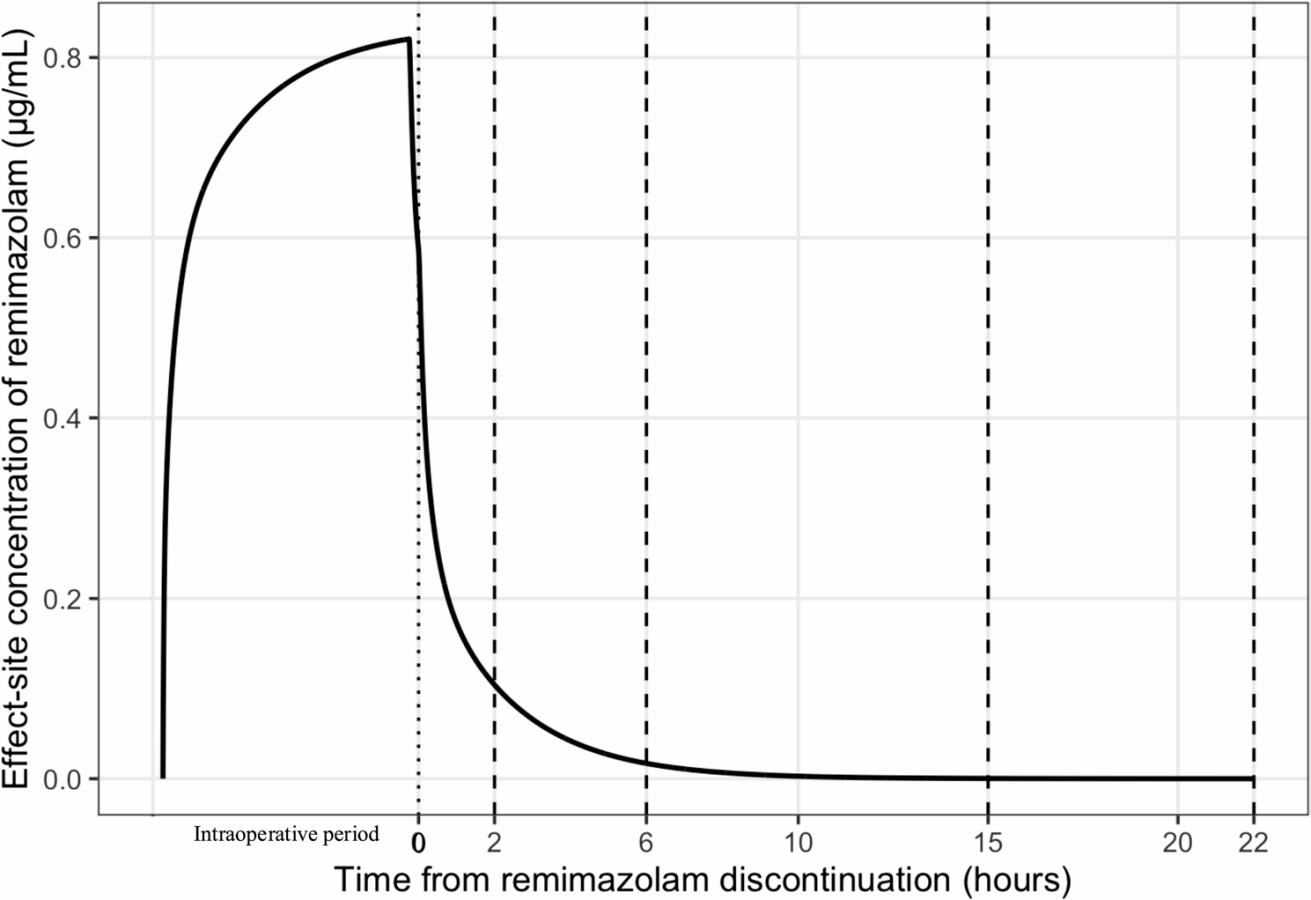
Simulated effect-site concentration-time profile during the perioperative period using the Masui model. According to this Masui model, dose regimens designed to maintain an effect-site concentration of 0.6–1.0 µg/mL during steady-state maintenance of general anesthesia. In the present simulation, the effect-site concentration during the surgical period remained within this range, supporting that the achieved perioperative plasma level was appropriate for maintaining adequate anesthesia. The recovery of consciousness is considered to occur at effect-site concentrations below approximately 0.2 µg/mL. Thus, this simulation indicated that emergence should be achievable within at least one hour after discontinuation of remimazolam. This analysis was conducted using R version 4.0.2 (R Foundation for Statistical Computing, Vienna, Austria).

Our findings underscore that remimazolam should be considered as a possible cause of postoperative impaired consciousness requiring ICU admission after hepatectomy using the Pringle maneuver.

## Data Availability

The data discussed in this report are available from the corresponding author upon request.

## Abbreviations

ICU: intensive care unit
